# Factors Associated With Breast Cancer Preventive Behaviors Among Female Health Ambassadors in Khoy: An Analysis Based on the Protection Motivation Theory

**DOI:** 10.1155/ijbc/7656595

**Published:** 2026-07-13

**Authors:** Morad Ali Zareipour, Leila Mokhtari, Rahim Sharafkhani, Hamideh safaralizadeh, Mohammad Saeed Jadgal

**Affiliations:** ^1^ Department of Public Health, Khoy University of Medical Sciences, Khoy, Iran; ^2^ Department of Nursing, Khoy University of Medical Sciences, Khoy, Iran; ^3^ Health System Research Committee, Khoy University of Medical Sciences, Khoy, Iran; ^4^ Tropical and Communicable Diseases Research Center, Iranshahr University of Medical Sciences, Iranshahr, Iran, irshums.ac.ir; ^5^ Department of Public Health, Chabahar University of Medical Sciences, Chabahar, Iran

**Keywords:** breast cancer, health ambassadors, preventive behaviors, protection motivation theory

## Abstract

**Introduction and Objective:**

Breast cancer is the most prevalent malignancy among Iranian women, and early detection plays a pivotal role in reducing mortality. This study was aimed at investigating factors associated with breast cancer preventive behaviors among female health ambassadors in Khoy, using the protection motivation theory as the theoretical framework.

**Methods:**

This cross‐sectional analytical study was conducted among 160 female health ambassadors in Khoy in 2023. Data were collected using a standardized questionnaire based on protection motivation theory constructs, including knowledge, perceived susceptibility, perceived severity, self‐efficacy, response efficacy, response costs, fear, and protection motivation. Data were analyzed using Stata Version 18, applying backward linear regression to identify factors associated with breast cancer preventive behaviors.

**Results:**

Among demographic variables, university education (*B* = 2.05, *p* = 0.008) and employment status (*B* = 1.58, *p* = 0.002) were significantly associated with breast cancer preventive behaviors. Among protection motivation theory constructs, fear (*B* = 0.32, *p* < 0.001) showed the strongest association, followed by protection motivation (*B* = 0.25, *p* = 0.007), knowledge (*B* = 0.10, *p* = 0.002), and perceived severity (*B* = 0.09, *p* < 0.001); all variables were significantly associated with preventive behaviors. The final model explained 65.6% of the variance in preventive behaviors (*R* ^2^ = 0.656, adjusted *R* ^2^ = 0.639, *p* < 0.001).

**Conclusion:**

The findings support the applicability of the protection motivation theory in explaining breast cancer preventive behaviors among female health ambassadors in Khoy City. Theory‐based educational interventions, potentially tailored for similar demographic groups characterized by lower education levels and unemployment, may help strengthen preventive practices. However, due to the cross‐sectional design of this study and its focus on a specific population in a single city, the observed relationships should be interpreted strictly as associations rather than causal effects, and these results cannot be generalized to all Iranian women. These findings highlight the potential role of community health educators in developing targeted community‐based prevention programs for specific at‐risk populations.

## 1. Introduction

Breast cancer remains the most frequently diagnosed malignancy among women and a leading cause of cancer‐related mortality worldwide. Globally, an estimated 2.3 million new cases are diagnosed each year, representing approximately 15%–25% of all cancers affecting women [1, 2]. The disease accounts for more than 627,000 deaths annually, with nearly 80% of these fatalities occurring in low‐ and middle‐income countries [3]. In the Middle East region, the burden of breast cancer is increasing, and projections suggest that the number of new cases may double by 2030 [4].

In Iran, breast cancer constitutes a major public health concern and is the most common cancer among women. National standardized data indicate an incidence rate of approximately 40–43 cases per 100,000 population [5]. The mean age at diagnosis among Iranian women is around 47 years, nearly a decade younger than that reported in many developed countries [6]. Notably, about 20% of affected women in Iran are younger than 40 years [6]. Breast cancer is currently ranked as the fifth leading cause of cancer‐related mortality in the country [6].

Given its high prevalence and substantial health burden, early detection and preventive strategies have been prioritized within national health programs. Primary prevention is widely recognized as a cost‐effective approach to reducing cancer‐related mortality [7]. Increasing women′s awareness and encouraging engagement in preventive behaviors, including regular screening practices, are therefore essential. Standard screening methods include clinical breast examination performed by healthcare professionals, mammography, and regular breast self‐examination. Breast self‐examination, in particular, is a simple, low‐cost, and accessible method that may facilitate early identification of suspicious breast changes [8].

Despite the documented benefits of screening, evidence indicates that many Iranian women do not consistently adhere to recommended preventive practices. A national survey reported that only about half of women have ever performed breast self‐examination, while more than 70% have never undergone a clinical breast examination by a physician [9, 10]. Understanding the psychological and contextual factors that influence women′s engagement in preventive behaviors is therefore crucial.

Protection motivation theory (PMT) provides a well‐established theoretical framework for examining health‐related behaviors [11]. According to PMT, protective behaviors are influenced by two cognitive appraisal processes: threat appraisal (including perceived susceptibility, perceived severity, and fear) and coping appraisal (including self‐efficacy and response efficacy) [12]. The theory posits that individuals who perceive a higher level of threat and have greater confidence in their ability to perform effective preventive actions are more likely to develop stronger protection motivation and, consequently, engage in protective behaviors [11, 12].

Key strategies for breast cancer prevention include adherence to regular screening programs (clinical examinations and periodic mammography), monthly breast self‐examination performed correctly, and maintenance of a healthy lifestyle characterized by balanced nutrition and regular physical activity [10, 13]. When implemented collectively, these measures substantially contribute to risk reduction and early diagnosis. Studies applying PMT have consistently reported that constructs such as fear, perceived severity, response efficacy, self‐efficacy, response costs, and protection motivation are significantly associated with breast cancer screening behaviors, including clinical breast examination, breast self‐examination, and mammography. Higher levels of threat appraisal and coping appraisal have been linked to greater engagement in preventive and screening practices among women. These findings are consistent with the present study and further support the relevance of PMT constructs in explaining variations in breast cancer preventive behaviors [3, 14, 15].

Women also play a pivotal role in promoting family health. Within Iran′s healthcare system, each household designates a “health ambassador,” typically a literate female family member who serves as a community health volunteer responsible for disseminating health information and reinforcing preventive practices among family members [16]. Health ambassadors serve as a critical link between healthcare centers and households, facilitating communication and modeling healthy behaviors within the family setting [17].

Given the strategic position of health ambassadors in influencing community health behaviors, examining breast cancer preventive practices among this group is of particular importance. However, limited research has specifically addressed breast cancer preventive behaviors among female health ambassadors. Therefore, the present study was aimed at investigating the factors associated with breast cancer preventive behaviors among female health ambassadors in Khoy, using the PMT as the guiding framework.

## 2. Materials and Methods

### 2.1. Study Design

This research is a cross‐sectional study of a descriptive‐analytical nature. The study population consisted of all health ambassadors affiliated with the health centers of Khoy City. The inclusion criteria were being a female health ambassador with an active record at health centers or health posts, being 15 years of age or older, and providing informed consent to participate in the study. The exclusion criteria included a previous or current diagnosis of breast cancer and incomplete submission of the questionnaire.

### 2.2. Sampling Method

The sample size for this study was determined using a formula for estimating a population mean, considering the standard deviation of breast cancer preventive behaviors reported in a previous relevant study [5]. Based on a standard deviation of 3.73 for these behaviors, a 95% confidence level (*α* = 0.05), and a desired margin of error (*d* = 0.6), the required sample size was calculated to be 149 individuals. To account for potential attrition and to ensure sufficient statistical power for the planned analyses (e.g., correlation and regression), the final sample size was set at 160 participants.

n=Z12−a/2×σ2 d2

The sampling procedure was conducted as follows: First, the city of Khoy was divided into two municipal districts (based on the municipality′s division). From the existing healthcare centers in each district, four centers were selected using simple random sampling, resulting in a total of eight selected healthcare centers.

Subsequently, from the list of health ambassadors affiliated with each selected center, 20 individuals were chosen by simple random sampling. The selected health ambassadors were contacted by the research team via the telephone numbers available at the health centers. They were invited to participate in the study and, if willing, were scheduled for a visit. A total of 160 eligible participants were invited to participate in the study, and all completed the questionnaire, resulting in a response rate of 100%. Therefore, the risk of nonresponse bias is considered minimal.

### 2.3. Data Collection Tools

The questionnaires were administered and completed in private rooms at the respective healthcare centers to ensure confidentiality and a comfortable environment for the participants. The data collection tools consisted of a questionnaire comprising three main sections: a demographic characteristics section, a knowledge assessment questionnaire, and a questionnaire based on the PMT.

#### 2.3.1. Demographic Characteristic Assessment

The demographic questionnaire included items on age (categorized as 15–25, 25–35, 35–45, and more than 45 years), marital status (single, married, and widow or divorced), education level (primary or high school, diploma, and university), job status (housewife and employee), family history of cancer (yes and no), and economic situation. Economic status was assessed using a single self‐report question: “How do you evaluate your economic status compared to the community?” with response options of “weak,” “moderate,” or “good,” reflecting participants’ perceived economic status relative to the general population rather than objective income‐based criteria.

#### 2.3.2. Questionnaires: Knowledge and PMT Constructs

The data collection tools included three questionnaires: a demographic questionnaire, a knowledge questionnaire, and a PMT questionnaire. The validity and reliability of the questionnaires were confirmed by Khodayarian et al. [18], who reported a Content Validity Index of 0.92 and a Cronbach′s alpha of 0.9 for the overall instrument, with individual construct reliabilities ranging from 0.75 to 0.88. In the present study, the reliability of the questionnaire constructs was further assessed using Cronbach′s alpha (coefficients presented in Table 1) after administration to a sample of 30 health ambassadors. Knowledge was assessed using items with *Yes*, *No*, and *I don’t know* response options, which were assigned scores of 2, 0, and 1, respectively. The total score for this section ranged from 0 to 24. The constructs of the PMT, namely, perceived susceptibility, perceived severity, intrinsic reward, response efficacy, self‐efficacy, response cost, fear, and protection motivation, were assessed using a 5‐point Likert scale ranging from *Strongly Agree* to *Strongly Disagree*. The total score ranged from 8 to 40 for perceived susceptibility. For perceived severity, intrinsic reward, response efficacy, self‐efficacy, and response cost, the total score ranged from 7 to 35. The total score ranged from 5 to 25 for fear [19] and from 4 to16 for protection motivation.

**Table 1:** Number of items, mean, standard deviation, minimum and maximum scores, and Cronbach′s alpha for the constructs of the research questionnaire.

Variable	No. of items	Mean	Std. dev.	Min	Max	Cronbach′s *α*
Knowledge	12	20.06	5.82	12	36	0.91
Perceived susceptibility	8	22.86	6.58	8	40	0.83
Perceived severity	7	21.50	9.26	7	35	0.85
Rewards	7	22.10	5.19	7	35	0.78
Response efficacy	7	20.47	5.63	12	35	0.84
Self‐efficacy	7	25.46	5.49	14	54	0.75
Response costs	7	24.61	5.57	7	35	0.88
Fear	5	12.86	4.93	5	25	0.78
Protective motivation	4	9.72	2.28	5	15	0.74
Preventive behaviors	6	10.05	3.79	12	30	0.84

Preventive behaviors were evaluated using items on a 5‐point frequency scale from *Always* to *Never*, with scores assigned from 5 to 1, respectively. The total score for this section ranged from 6 to 30.

### 2.4. Data Analysis

Upon completion of the questionnaires by the study participants, the data were collected and prepared for analysis. The dataset was checked for missing values before analysis. No missing data were identified, as all questionnaires were completed in full at the time of collection. Prior to conducting the statistical analyses, the assumptions underlying parametric tests were examined. The normality of the data distribution was assessed using the Shapiro–Wilk test, and the homogeneity of variances was evaluated using Levene′s test. The results indicated that the assumptions for applying parametric tests were satisfied. The statistical analyses employed included ANOVA, independent samples *t*‐test, and ordinal logistic regression using the backward elimination method. For the regression analysis, variables with a *p* value of less than 0.2 in the univariate analysis were entered into the initial model. In each subsequent step, the variable with the highest *p* value that did not show a significant effect on the outcome was removed from the model. Variables with a *p* value of less than 0.05 were retained in the final model. In addition, variables considered theoretically important based on previous literature were retained in the model to control for potential confounding effects, even if they were not statistically significant. All statistical analyses were performed using STATA software, Version 18.

### 2.5. Ethical Considerations

Ethical approval was obtained from the Human Research Ethics Committee at the Khoy University of Medical Sciences. All study participants provided written informed consent. Confidentiality and anonymity were ensured. All procedures performed in studies involving human participants were in accordance with the ethical standards of the institutional and/or national research committee and with the 1964 Helsinki Declaration. Also, this study was approved by the Research Ethics Committee of Khoy University of Medical Sciences (Ethics Code: IR.KHOY.REC.1402.013).

## 3. Results

The results showed that the largest age group among the participants was 35–45 years (67 participants, 41.9%). Most participants were married (132, 82.5%). In terms of education, the largest proportion had a university degree (54, 22.5%). Regarding occupation, the majority were homemakers (110, 68.8%). Additionally, most participants reported no family history of cancer (149, 93.1%), and the majority had a moderate economic status (100, 62.5%) (Table 2).

**Table 2:** Demographic status of health ambassadors participating in the study.

Variable	*N*(%)
Age	
15–25	28 (17.5%)
25–35	47 (29.4%)
35–45	67 (41.9%)
More than 45	18 (11.2%)
Marital status	
Single	21 (13.1%)
Married	132 (82.5%)
Widow or divorced	7 (4.4%)
Education	
Primary or high school	56 (8.1%)
Diploma	50 (12.5%)
University	54 (22.5%)
Job	
Housewife	110 (68.8%)
Employee	50 (31.2%)
Family cancer	
Yes	11 (6.9%)
No	149 (93.1%)
Economic situation	
Weak	32 (20.0%)
Moderate	100 (62.5%)
Good	28 (17.5%)

The results demonstrated significant associations between several demographic characteristics and breast cancer preventive behaviors. Marital status (*p* < 0.001), education level (*p* = 0.001), and occupation (*p* < 0.001) were significantly associated with preventive behaviors. Married participants reported the highest mean score (19.77 ± 3.59). Participants with a high school diploma (19.92 ± 3.14) and those with a university degree (19.91 ± 3.86) had significantly higher mean scores compared with individuals with primary education. Employed participants also showed higher mean scores than homemakers (21.97 ± 5.38 vs. 18.32 ± 2.87). No statistically significant associations were found between preventive behaviors and age (*p* = 0.680), family history of cancer (*p* = 0.170), or economic status (*p* = 0.180) (Table 3).

**Table 3:** Association between demographic characteristics and mean scores of breast cancer preventive behaviors.

Variable	*M* *e* *a* *n* ± *S* *D*	*p*value
Age		
15–25	18.43 ± 2.89	0.68 ^∗^
25–35	19.32 ± 3.72	
35–45	19.25 ± 4.22	
More than 45	18.56 ± 3.63	
Marital status		
Single	14.48 ± 1.08	0.0001 > ^∗^
Married	19.77 ± 3.59	
Widow or divorced	19.0 ± 2.83	
Education		
Primary or high school	15.58 ± 1.86	0.001 ^∗^
Diploma	19.92 ± 3.14	
University	19.91 ± 3.86	
Job		
Housewife	18.32 ± 2.87	0.0001 > ^∗^
Employee	21.97 ± 5.38	
Family cancer		
Yes	17.55 ± 2.77	0.17 ^∗∗^
No	19.16 ± 3.84	
Economic situation		
Weak	19.22 ± 3.75	0.18 ^∗^
Moderate	18.68 ± 3.48	
Good	20.18 ± 4.7	

*Note:* *p* value from one‐way ANOVA for comparisons among ≥ 3 groups and independent *t*‐test for two‐group comparisons. Post hoc pairwise comparisons (Tukey′s HSD) were conducted for variables with significant ANOVA results (marital status, education, and job). Detailed results of post hoc analyses are reported in the text.

^∗^ ANOVA.

^∗∗^ Independent samples *t*‐test.

As shown in Table 3, one‐way ANOVA revealed significant associations between marital status, education level, job status, and the mean scores of breast cancer preventive behaviors (*p* < 0.05). To further explore these differences, post hoc analyses using Tukey′s HSD test were conducted. Regarding marital status, post hoc comparisons indicated that married individuals (19.77 ± 3.59) and widowed or divorced individuals (19.00 ± 2.83) had significantly higher preventive behavior scores than single individuals (14.48 ± 1.08), with *p* < 0.001 for both comparisons. However, no significant difference was observed between married and widowed or divorced groups (*p* = 0.34). Concerning education level, participants with a diploma (19.92 ± 3.14) and those with a university education (19.91 ± 3.86) scored significantly higher than those with primary or high school education (15.58 ± 1.86), with *p* < 0.001 for both comparisons. No significant difference was found between diploma and university groups (*p* = 0.98). For job status, employees (21.97 ± 5.38) had significantly higher preventive behavior scores compared to housewives (18.32 ± 2.87), with *p* < 0.001. For age, family history of cancer, and economic situation, no significant differences were observed (*p* > 0.05), and therefore, post hoc tests were not performed for these variables.

Table 1 presents the characteristics of the key variables of the PMT. The mean scores of the study variables were as follows: knowledge (M = 20.06), perceived susceptibility (M = 22.86), perceived severity (M = 21.50), intrinsic rewards (M = 22.10), response efficacy (M = 20.47), self‐efficacy (M = 25.46), response costs (M = 24.61), fear (M = 12.86), protection motivation (M = 9.72), and preventive behaviors (M = 10.05).

Pearson′s correlation analysis among the constructs of PMT and breast cancer preventive behaviors revealed significant positive correlations among most theoretical constructs. Perceived severity, perceived susceptibility, response efficacy, and self‐efficacy were positively correlated with each other and with protection motivation. In contrast, response costs showed significant negative correlations with several PMT constructs, including knowledge, perceived severity, response efficacy, self‐efficacy, and fear. Preventive behaviors were positively correlated with most PMT constructs, including knowledge, perceived susceptibility, perceived severity, response efficacy, self‐efficacy, fear, and protection motivation. The strongest correlation was observed between fear and preventive behaviors (*r* = 0.63, *p* < 0.001) (Table 4).

**Table 4:** Pearson′s correlation coefficients between the constructs of PMT and breast cancer preventive behaviors.

Variables	(1)	(2)	(3)	(4)	(5)	(6)	(7)	(8)	(9)	(10)
(1) Knowledge	1.00									
*p*	—									
(2) Perceived susceptibility	0.25	1.00								
*p*	0.00	—								
(3) Perceived severity	0.27	0.47	1.00							
*p*	0.00	0.00	—							
(4) Intrinsic rewards	0.06	0.13	0.31	1.00						
*p*	0.45	0.10	0.00	—						
(5) Response efficacy	0.12	0.21	0.43	0.33	1.00					
*p*	0.12	0.01	0.00	0.00	—					
(6) Self‐efficacy	0.09	0.02	0.36	0.24	0.44	1.00				
*p*	0.24	0.82	0.00	0.00	0.00	—				
(7) Response costs	−0.26	−0.15	−0.18	−0.30	−0.34	−0.31	1.00			
*p*	0.00	0.06	0.02	0.00	0.00	0.00	—			
(8) Fear	0.24	0.34	0.28	0.00	0.21	0.08	−0.19	1.00		
*p*	0.00	0.00	0.00	0.96	0.01	0.29	0.01	—		
(9) Protective motivation	0.25	0.19	0.28	−0.03	0.20	0.01	−0.02	0.34	1.00	
*p*	0.00	0.02	0.01	0.72	0.01	0.86	0.79	0.00	—	
(10) Preventive behaviors	0.41	0.44	0.55	0.01	0.34	0.17	−0.11	0.63	0.45	1.00
*p*	0.00	0.00	0.00	0.21	0.00	0.03	0.17	0.00	0.00	—

Backward linear regression analysis was performed to identify factors associated with breast cancer preventive behaviors (Tables 5 and 6). Given the potential risk of overfitting in backward regression models, the final model was interpreted with caution and in light of theoretical relevance. In addition, regression diagnostics were conducted. Multicollinearity was assessed using variance inflation factor (VIF) and tolerance statistics, and no evidence of problematic multicollinearity was observed. Furthermore, the assumptions of linearity, normality of residuals, and homoscedasticity were examined and found to be adequately satisfied.

**Table 5:** Backward linear regression analysis of factors associated with breast cancer preventive behaviors.

Variable	*B*(unstandardized)	Std. err.	*T*	*p* > |*t*|	95% CI	VIF	Tolerance
Education	1	—	—	—	—	—	—
Primary or high school	1.08	0.83	1.30	0.20	[−0.56 to 2.72]	1.45	0.69
Diploma	2.52	0.83	3.03	0.003	[0.88–4.16]	1.52	0.66
University	2.05	0.77	2.68	0.008	[0.54–3.57]	1.48	0.68
Job	1	—	—	—	—	—	—
Employee	1.58	0.51	3.11	0.002	[0.57–2.58]	1.22	0.82
Knowledge	0.104	0.03	3.11	0.002	[0.04–0.17]	1.35	0.74
Perceived severity	0.088	0.02	3.68	< 0.001	[0.41–0.14]	1.62	0.62
Fear	0.32	0.04	7.78	< 0.001	[0.24–0.401]	1.78	0.56
Protection motivation	0.25	0.08	2.74	0.007	[0.07–0.42]	1.85	0.54
Constant	6.36	1.06	5.98	< 0.001	[4.26–8.46]	—	—

**Table 6:** ANOVA and Summary Statistics for the Multiple Linear Regression Analysis of Factors Associated with Breast Cancer Preventive Behaviors (N = 160).

Source	SS	df	*N* *u* *m* *b* *e* *r* of *o* *b* *s* = 160	*F*(8,151) = 36.10
MS				
Model	1498.19	8	187.27	Prob > *F* < 0.001
Residual	783.42	151	5.19	*R* − squared = 0.656
Total	2281.60	159	14.36	Adj *R* − squared = 0.639
				Root MSE = 2.28

*Note:* *B*, unstandardized regression coefficient. Regression assumptions, including linearity, homoscedasticity, and normality of residuals, were adequately met. The absence of significant multicollinearity was indicated by VIF values less than 10 and tolerance values greater than 0.1.

Abbreviations: CI, confidence interval; VIF, variance inflation factor.

Among the demographic variables, educational status was significantly associated with breast cancer preventive behaviors. Compared with women in the reference group, those with a diploma had significantly higher preventive behavior scores (*B* = 2.52; 95% CI: 0.88–4.16; *p* = 0.003), and those with university education also had significantly higher scores (*B* = 2.05; 95% CI: 0.54–3.57; *p* = 0.008). Occupational status was also significantly associated with preventive behaviors, with employed women reporting higher scores than homemakers (*B* = 1.58; 95% CI: 0.57–2.58; *p* = 0.002).

Among the PMT constructs, fear showed the strongest association with breast cancer preventive behaviors (*B* = 0.32; 95% CI: 0.24–0.40; *p* < 0.001), followed by protection motivation (*B* = 0.25; 95% CI: 0.07–0.42; *p* = 0.007), knowledge (*B* = 0.10; 95% CI: 0.04–0.17; *p* = 0.002), and perceived severity (*B* = 0.09; 95% CI: 0.04–0.14; *p* < 0.001); all were significantly and positively associated with breast cancer preventive behaviors. All reported coefficients are unstandardized regression coefficients (*B*), representing the expected difference in the preventive behavior score associated with a one‐unit increase in each variable while other variables are held constant. The final regression model explained 65.6% of the variance in breast cancer preventive behaviors (*R* ^2^ = 0.656; adjusted *R* ^2^ = 0.639) and was statistically significant (*F*(8,151) = 36.10; *p* < 0.001).

## 4. Discussion

The present study identified educational and occupational status as factors significantly associated with breast cancer preventive behaviors among female health ambassadors. Higher educational attainment was associated with greater engagement in preventive behaviors. This finding is consistent with previous research indicating that lower education acts as a barrier to breast self‐examination, clinical examination, and mammography uptake [20–22]. Education may facilitate preventive behavior through several mechanisms, including improved health literacy, greater access to reliable information, enhanced critical appraisal skills, and increased autonomy in health‐related decision‐making. These cognitive and structural advantages may help individuals better understand cancer risk and navigate screening services.

Employment status was also positively associated with preventive behaviors, a finding supported by prior national and international studies [6, 23]. Employment may facilitate engagement in screening through financial independence, access to health insurance, broader social networks, and greater exposure to workplace health promotion programs. In addition, employed health ambassadors may serve as important channels for disseminating preventive health information within their professional and community networks, potentially amplifying the broader public health impact.

The findings also support the usefulness of PMT in understanding breast cancer preventive behaviors. The PMT constructs were significantly associated with preventive behaviors, highlighting the relevance of both threat appraisal and coping appraisal processes. Among the PMT constructs, fear showed the strongest association with preventive behaviors. Consistent with previous evidence [24–26], moderate and cognitively processed fear may be associated with greater protective motivation when individuals simultaneously perceive effective coping strategies. Within the PMT framework, fear may function not only as an emotional response but also as a factor that may heighten risk perception and encourage adaptive coping when accompanied by high self‐efficacy and response efficacy.

Protection motivation itself was also significantly associated with preventive behaviors, consistent with prior studies suggesting that the cognitive appraisal of threat combined with confidence in coping ability is closely related to behavioral engagement [1, 3, 27]. This supports the theoretical proposition that individuals are more likely to adopt preventive behaviors when they perceive the threat as meaningful and believe that the recommended action is both feasible and effective. Accordingly, interventions should strengthen coping appraisal components rather than relying solely on threat communication.

Knowledge was another factor significantly associated with preventive behavior, consistent with earlier studies demonstrating that informed individuals are more likely to participate in screening and early detection practices [11, 14, 28, 29]. Within PMT, knowledge serves as a foundational cognitive resource that facilitates accurate threat appraisal and evaluation of response options. However, knowledge alone may be insufficient; its behavioral relevance depends on whether it contributes to greater perceived severity, susceptibility, and confidence in one′s ability to act. Therefore, educational interventions should combine informational content with strategies that enhance self‐efficacy and response efficacy.

Perceived severity was also positively associated with preventive behaviors, in line with previous research [1, 30]. When individuals perceive breast cancer as a serious threat affecting survival, family roles, and quality of life, their motivation to engage in preventive actions may increase. Perceived severity therefore represents an important link between awareness and action; without a meaningful appraisal of consequences, factual knowledge may not translate into behavioral change. Accordingly, effective health education should move beyond simple information provision and foster a realistic and personally relevant understanding of disease impact.

Overall, these findings highlight the importance of integrating demographic considerations with theoretically grounded cognitive constructs when designing interventions. Tailored, literacy‐sensitive, and theory‐based educational strategies, particularly those that strengthen coping appraisal while appropriately addressing threat perception, may help promote breast cancer preventive behaviors.

### 4.1. Study Limitations

Despite the valuable insights provided by this study, several limitations should be considered. First, the cross‐sectional design limits the ability to draw causal inferences between the constructs of PMT and breast cancer preventive behaviors. Second, the study population consisted exclusively of female health ambassadors, which may restrict the generalizability of the findings to other groups of women with different sociodemographic characteristics. Third, the use of self‐reported questionnaires may introduce response bias, including social desirability bias. In addition, although efforts were made to recruit participants systematically, the possibility of selection bias cannot be entirely excluded. Future research using longitudinal or experimental designs and involving more diverse populations would help further examine and extend these findings.

### 4.2. Conclusion

The findings of this study support the applicability of the PMT in explaining breast cancer preventive behaviors among female health ambassadors in Khoy City, with the developed model accounting for 65.6% of the variance within this specific population.

The findings of this study support the applicability of the PMT in understanding breast cancer preventive behaviors among female health ambassadors in Khoy City, with the model accounting for 65.6% of the variance in preventive behaviors within this population.

These findings may have implications for community health education in similar settings. Community health educators may consider using this information when designing targeted educational interventions. Such interventions could emphasize the seriousness of breast cancer to strengthen protective motivation while also enhancing self‐efficacy through practical training in breast self‐examination and participation in screening programs. Educational strategies may also incorporate PMT constructs into individualized risk‐assessment counseling, use fear‐based messages cautiously to encourage preventive action without provoking excessive anxiety, and organize community‐based educational sessions aimed at reducing perceived barriers to screening.

By applying this theoretical framework, community health educators may be able to provide more structured and psychologically informed health education that could support women in adopting preventive behaviors. This approach may be particularly relevant for vulnerable groups in similar contexts, such as homemakers and women with lower educational levels. For these groups, tailored, theory‐based counseling and community programs may help translate awareness into preventive action.

## Funding

No funding was received for this manuscript.

## Conflicts of Interest

The authors declare no conflicts of interest.

## Data Availability

The data that support the findings of this study are available upon request from the corresponding author. The data are not publicly available due to privacy or ethical restrictions.
